# ^68^Ga-PSMA PET/CT in early relapsed prostate cancer patients after radical therapy

**DOI:** 10.1038/s41598-022-24688-3

**Published:** 2022-11-28

**Authors:** Mohamad Haidar, Alain S. Abi-Ghanem, Hicham Moukaddam, Malak El Jebai, Safaa Al Zakleet, Samir Al Rayess, Abdul Rahman Akkawi, Mutaz Kassas, Hani Tamim, Albert El Hajj, Enrique Estrada-Lobato, Medhat M. Osman, Ali Shamseddine

**Affiliations:** 1 Department of Diagnostic Radiology, Faculty of Medicine, American University of Beirut Medical Center, Beirut, Lebanon; 2 Department of Internal Medicine, University of Kansas School of Medicine-Wichita, Wichita, KS USA; 3 Clinical Research Institute, American University of Beirut, Beirut, Lebanon; 4 Division of Urology, Department of Surgery, American University of Beirut Medical Center, Beirut, Lebanon; 5 Nuclear Medicine and Diagnostic Imaging Section, Division of Human Health, International Atomic Energy Agency, Vienna, Austria; 6 Division of Nuclear Medicine, Department of Radiology, Saint Louis University Hospital, St. Louis, MO 63110 USA; 7 Department of Internal Medicine, Division of Hematology and Oncology, Faculty of Medicine, Naef K. Basile Cancer Institute, American University of Beirut Medical Center, Beirut, Lebanon

**Keywords:** Cancer, Diseases, Prostatic diseases, Cancer

## Abstract

Biochemical recurrence (BCR) of prostate cancer (PCa) occurs in about 25% of patients treated with radical prostatectomy (RP) and up to 45% in patients who receive external beam radiotherapy (RT). Early diagnosis of PCa recurrence is of high importance for successful salvage therapy. The aim of the present study is to analyze the efficacy of ^68^ Ga-PSMA PET/CT in detecting the presence of local and/or systemic disease in patients with a history of PCa who have BCR. A total of 52 PCa patients with BCR referred for ^68^ Ga-PSMA PET/CT were recruited from the American University of Beirut Medical Center between November 2017 and December 2019. We compared the performance of PSMA PET/CT to the results and clinical factors based on follow up: PSA, PSA kinetics, primary treatment, and Gleason score. The relationship between the PET/CT findings and clinical indicators of disease were assessed by univariate and multivariate logistic regression. From a total of 52 patients, 34 (65.4%) had positive PSMA-PET/CT scans. Among those, 8/34 (23.5%) received primary RT. For all patients with a positive PSMA-PET: the detection rate was 2/4 (50%) for PSA < 0.2, 5/10 (50%) for PSA 0.2–0.49, 3/6 (50%) for PSA 0.5–0.99, 6/12 (50%) for PSA 1–1.99, 8/9 (88.9%) for PSA 2–3.99, and 10/11 (90.9%) for PSA 4–10.

PSMA-PET/CT positivity was significantly associated with PSA level at time of PET scan, PSA doubling time, Gleason score and TNM staging. However, it did not show a significant correlation with radiotherapy as primary treatment, ongoing androgen deprivation therapy (ADT), time to relapse, and initial PSA before therapy. In our single center prospective trial, ^68^ Ga-PSMA PET/CT successfully detected the recurrence of PCa in patients with BCR. Scan positivity was significantly associated with PSA level at time of PET scan, PSA doubling time, Gleason score, and TNM staging. PSMA- PET/CT is a highly promising modality in the work up of patients with PCa in the setting of BCR for earlier detection of disease recurrence.

## Introduction

Prostate cancer (PCa) is the most common solid malignancy in men and the third leading cause of cancer-related death in western Europe and the United States^1,2^. Prostate Specific Antigen (PSA) is a widely used test for PCa screening. Biochemical Recurrence (BCR) is common across all modes of intervention, with a 10-year cumulative incidence of approximately 24% in patients who underwent radical prostatectomy (RP) and 43% in patients who received external beam radiotherapy^3^. In accordance with the nomograms, local recurrence in prostate bed after RP can be predicted with an accuracy of about 80% in patients with a BCR more than 3 years after RP, a PSA doubling time (PSAdt) > 11 months, Gleason Score (GS) < 7 and a pT3aN0 and pTxN1. In contrast, systemic recurrence can be predicted with an accuracy of about 80% in patients with a BCR distance of less than 1 year after RP, PSAdt of about 4–6 months, GS > 7 and stage of pT3b and pTxN1^4,5^. Despite a good sensitivity in distinguishing between local and distant recurrence, the nomograms do not provide information about either the real site of recurrence (lymph node vs. bone; pelvic vs. extra-pelvic) or the actual number of metastatic lesions. Moreover, most patients have features that can be compatible, in agreement with the nomograms, with both local and systemic recurrence. As such, targeted rescue therapies cannot be organized taking into consideration the possibility of risk supplied by only nomograms. Patients are therefore generally directed to a salvage radiotherapy (S-RT) of the prostate bed (suspicion of local recurrence) or to a systemic treatment with Androgen Deprivation Therapy (ADT) in case of suspicion of systemic recurrence. ADT + RT combination has also been used as standard treatment of BCR for locally advanced high-risk PCa^6,7^.

Conventional imaging methods, including computed tomography (CT), bone scintigraphy (BS) and magnetic resonance (MR), showed low accuracy values for restaging patients with BCR. During the last decade, nuclear medicine techniques such as positron emission tomography (PET), with ^11^C-choline and ^18^F-choline, were found to be more accurate than conventional diagnostic modalities for restaging patients with PCa showing BCR^8^, allowing the differentiation between a local recurrence confined to the pelvis from a systemic recurrence^9^. However, PET/CT with choline showed a suboptimal sensitivity when performed in patients with early BCR, i.e. patients with low serum PSA (< 0.2 ng/mL) after radical therapy^9^.

The optimal timing to implement rescue treatments (such as S-RT) for the best prognosis is when the extension of the disease is low, which is when serum PSA levels are first detectable after radical therapy^10^. In this context, there is a necessity for a diagnostic test potentially able to differentiate between initial or loco-regional from a systemic recurrence only manageable with palliative approaches.

One molecule used to target PCa is Prostate Specific Membrane Antigen (PSMA)^11^. PSMA is a membrane enzyme that is markedly expressed in PCa cells when compared with healthy prostate tissue^12^. The bond at the catalytic site of PSMA in its extracellular domain allows for the development of small-specific inhibitors that are internalized after ligand binding^13^. The use of ^68^ Ga-PSMA (Glu-NH-CO–NH-Lys- (Ahx)—[^68^ Ga (HBED-CC as a radiopharmaceutical for PET/CT imaging has proven to be more accurate in the diagnosis of early disease recurrence when compared to ^18^F-choline PET/CT^14,15^. These studies have also demonstrated a better tumor to background ratio (TBR) with ^68^ Ga-PSMA PET/CT compared to ^18^F-choline PET/CT in identifying suspicious lesions for relapse^13^. Further studies conducted on larger patient populations with BCR after radical therapy have shown an excellent diagnostic ability of ^68^ Ga-PSMA PET/CT in restaging patients with BCR, even when serum PSA values were very low^16–18^.

In recent months, the use of this tracer has been a subject of growing interest in the scientific community^16–21^. This radiopharmaceutical had also shown high specificity (> 90%) in studies using histological analysis as a reference standard for validation of PET results^16,19,21^. Finally, none of the studies in the literature had reported adverse events or clinically detectable pharmacological effects occurring concurrently or after executing the PET/CT ^68^ Ga-PSMA. For the reasons explained above, PSMA can be an excellent molecular target for the development of radiotracers for PET/CT imaging that can detect early disease relapse.

## Objectives

The primary aim of this study was to evaluate the detection rate, accuracy, and positivity rate of ^68^ Ga-PSMA PET/CT in detecting the presence of local and/ or systemic disease in patients with treated PCa and evidence of BCR. In addition, we aim to compare the performance of PSMA PET/CT to the results and clinical factors (GS, PSA levels, PSA kinetics, TNM staging) used in the normal care pathway.

## Materials and methods

### Study design

This study was done prospectively in patients with PCa at the American University of Beirut Medical Center, Lebanon. All patients had detectable serum PSA post primary therapy of PCa with either RP or RT. Fifty-two patients have been recruited into this study based on well-defined inclusion and exclusion criteria (Table 1).

**Table 1:** Inclusion and Exclusion Criteria.

Inclusion criteria	Exclusion criteria
Age > 35 years	Age < 35 years
Histopathology proven Prostatic Adenocarcinoma	History of any malignancy other than Prostate Cancer except for non-melanoma skin cancer
Previous Primary Treatment of Prostate Carcinoma with radical Prostatectomy or Radiotherapy	History of Paget’s disease
Biochemical Recurrence defined as After RP, a serum PSA level over 0.2 ng/ml confirmed by two subsequent consecutive measurements After RT, an absolute increase in PSA level of 2 ng/ml above nadir	Patients not treated with radical therapy (RP or EBRT)

All patients that were undergoing drug-use cures in the normal care pathway were evaluated. Eligible patients who participated in the study signed the informed consent before undergoing the ^68^ Ga-PSMA PET/CT scans.

Based on the European Association of Urology guidelines, patients were assigned to either local and/or systemic therapy^4^. Patients were then followed longitudinally with collection of clinical and biochemical data in 3-month intervals during the first year (T5-T6-T7-T8) and six months during the second year of follow-up (T9-T10). The duration of the enrollment of samples was twenty-four months. The duration of follow-up and clinical data collection for each individual patient was twenty-four months. This study was approved by the Institutional Review Board of American University of Beirut, and we confirm that all research was performed in accordance with relevant guidelines/regulations.

### 68 Ga-PSMA PET/CT imaging

Since this is a non-randomized study, all patients had a PET/CT scan with ^68^ Ga-PSMA. The radiopharmaceutical study of the tracer ^68^ Ga-PSMA are synthesized at the radiology pharmaceutical laboratory of AUBMC. The PET/CT scans were conducted with the following technical standards:

Good Manufacturing Practice (GMP) certified ^68^ Ga Radiolabelling Kit by Isotope Technologies Garching (Munich, Germany) and single-use sterile cassette by ABX Advanced Biochemical Compounds (ABX) (Radeberg, Germany) were labeled to 10 mg PSMA-11. Each patient received 65 to 178 megabecquerel (MBq) (mean 113.3 ± 21.2 MBq) (1.76–4.81 millicurie [mCi]; mean 3.06 ± 0.57 mCi) of ^68^ Ga-PSMA 11 intravenously. Sixty minutes post-injection of the radiotracer, whole-body images were acquired in supine position using a Philips Gemini TF 16 PET/CT scanner. Adverse reactions to the radiotracer were not experienced in any patient.

Two board-certified specialists with 23 years of experience in nuclear medicine and 7 years of experience in reporting ^68^ Ga PSMA PET/CT scans, reviewed all scans. The viewing and the processing of the images were made using a semi- quantitative workstation.

Images were interpreted with the dedicated commercially available software IntelliSpace Portal 8.0 by Philips Healthcare, which were displayed simultaneously as PET, CT, and PET/CT fusion series in axial cuts, sagittal and coronal, and 3D MIP (maximum intensity projection) reconstructions. Semi-quantitative SUVmax were measured in the lesions with higher uptake in the prostate bed, nodal, and extra-nodal metastasis.

All suspicious lesions with tracer uptake above the surrounding background activity and not conforming to benign or known pitfalls of ^68^ Ga-PSMA PET/CT findings were considered as sites of disease recurrence. Indeterminate PSMA findings were analyzed in light of clinical follow up, including PSA, imaging and response to therapy. The gold standard was PSA, imaging, and response to therapy.

### Statistical analysis

The demographic and clinical variables were arranged using descriptive analysis. The comparison between patients with positive and negative PSMA PET/CT results was performed using the t-test. The relationship between the PET/CT results and clinical indicators of disease status were assessed by univariate and multivariate logistic regression methods. The data was analyzed using statistical package SPSS version 21 for Windows.

### Consent to participate

Took approval from Institutional Review Board (IRB) of American University of Beirut. irb@aub.edu.lb.

## Results

### Patients’ characteristics

A total of 52 PCa patients with BCR referred for PSMA PET/CT between November 2017 and December 2019 were enrolled: 42 (80.8%) patients were treated initially with RP while 10 (19.2%) were managed with RT. The median time from PCa diagnosis to BCR was 41 months. At the time of PET/CT, 21 (40.3%) patients were ongoing ADT and 26 (50%) presented with a PSAdt ≤ 10 months. Table 2 shows the patients’ characteristics based on PSMA PET/CT results.

**Table 2:** Patients’ characteristics based on PSMA PET/CT results.

		PSMA-PET		*p*-value
		Negative N = 18	Positive N = 34	
Age	Mean ± SD	69.5 ± 7.4	72.3 ± 7.7	0.21
	Mean ± SD	1.15 ± 1.4	3.34 ± 3.2	0.006
PSA at PSMA/PET	< 0.2	2 (11.1%)	2 (5.9%)	
	0.2–0.49	5 (27.8%)	5 (14.7%)	0.09
	0.5–0.99	3 (16.7%)	3 (8.8%)	
	1–1.99	6 (33.3%)	6 (17.6%)	
	2–3.99	1 (5.6%)	8 (23.5%)	
	4–10	1 (5.6%)	10 (29.4%)	
PSA doubling time	Mean ± SD	8.6 ± 2.6	6.5 ± 2.5	0.006
	≤ 10	13 (72.2%)	32 (93.9%)	0.08
	> 10	5 (27.8%)	2 (6.1%)	
Baseline PSA before therapy	Mean ± SD	14.1 ± 15.6	18 ± 20.2	0.28
	< 10	10 (55.6%)	16 (47.1%)	
	10–20	5 (27.8%)	10 (29.4%)	0.93
	> 20	3 (16.7%)	8 (23.5%)	
TNM	T1	2 (11.1%)	3 (8.8%)	
	T2	13 (72.2%)	13 (38.2%)	0.03
	T3	3 (16.7%)	18 (52.9%)	
Time to relapse	Mean ± SD	47.4 ± 41.7	37.5 ± 45.6	0.21
Management with ADT	No ADT	14 (77.8%)	17 (50%)	0.08
	ADT	4 (22.2%)	17 (50%)	
Primary therapy	Primary radiotherapy	2 (11.1%)	8 (23.5%)	0.46
	Radical Prostatectomy	16 (88.9%)	26 (76.5%)	

The median age of the patients was 71.3 years: 41 (78.8%) men fulfilled eligibility based on having PSA < 4 ng/mL while 11 (21.2%) presented PSA concentration between 4 and 10 ng/mL with negative conventional imaging.

### 68 Ga-PSMA PET/CT imaging

**Figure 1:**
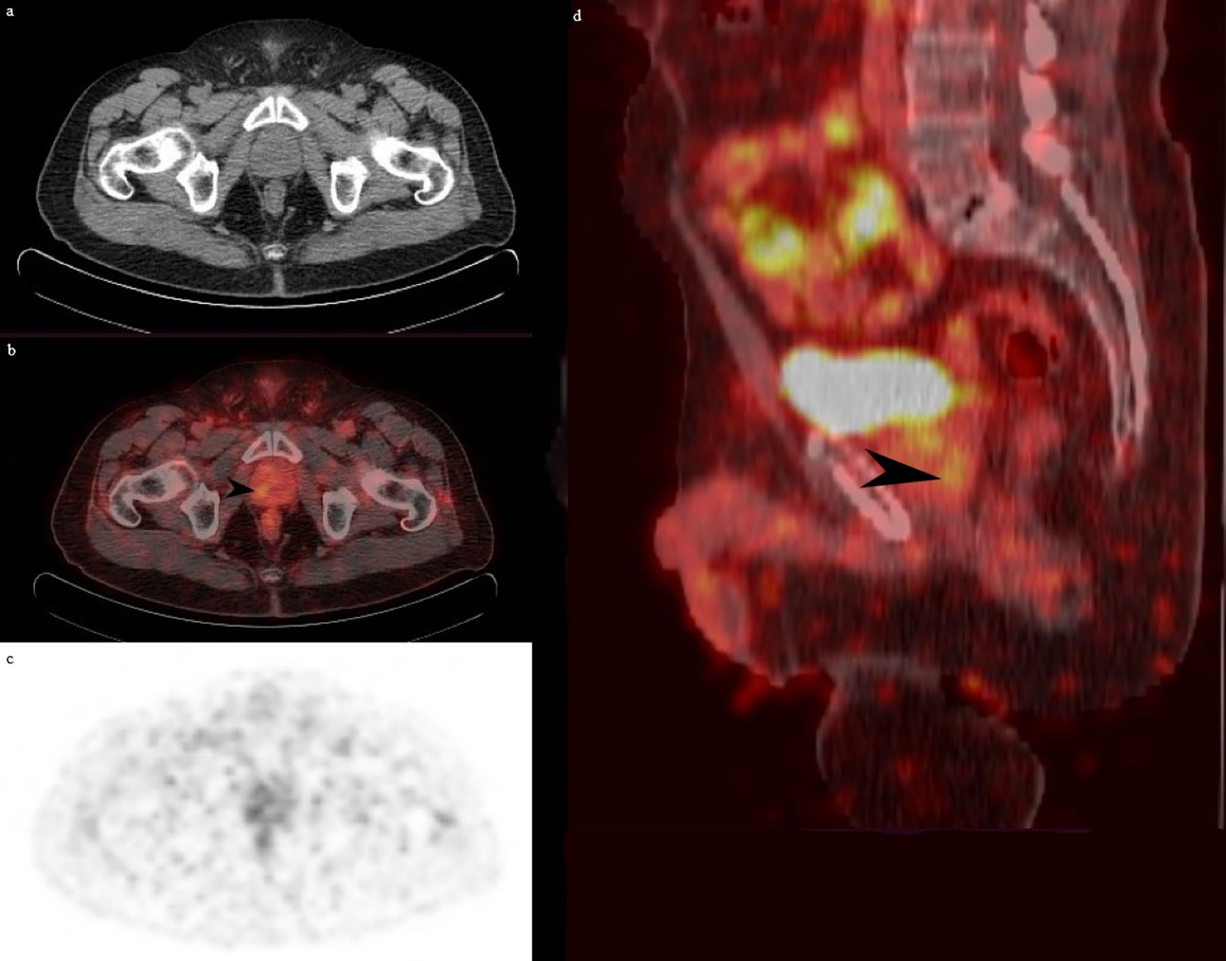
A 71-year-old patient on ADT and status post EBRT 60 Gray in 20 fractions. PSA at time of acquisition = 9.5 ng/mL. Axial CT (**a**) image of the prostate bed is shown. Fused PET/CT (**b**) shows focus of increased radiotracer uptake in the right peripheral zone of the prostate gland at mid-gland (black arrow) in keeping with disease recurrence. PET image is shown (**c**). Sagittal fused PET/CT (**d**) shows the described lesion (black arrow).

**Figure 2:**
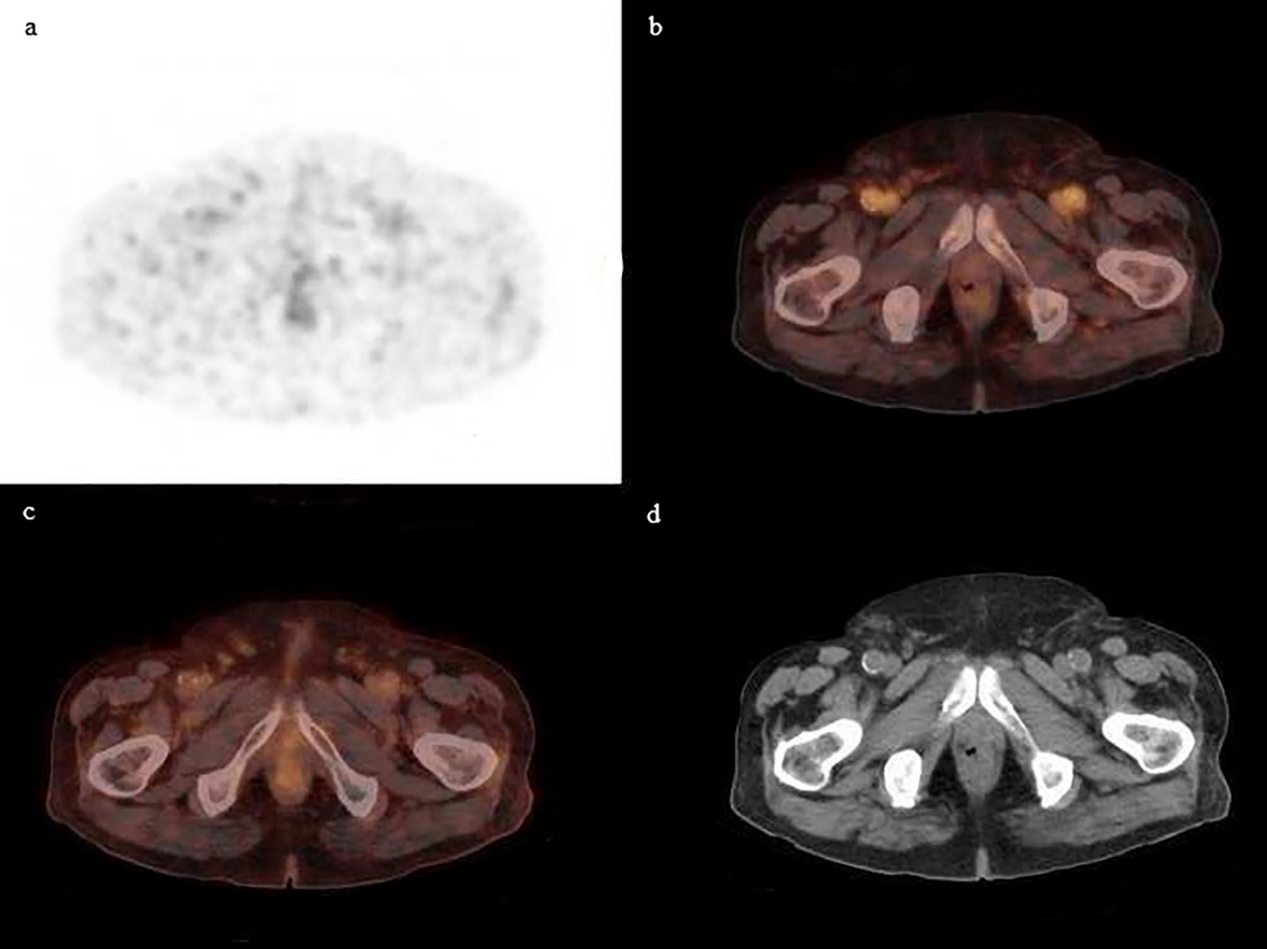
A 80-year-old patient status post radical prostatectomy. PSA level at time of acquisition: 1.38 ng/mL. Whole body ^68^ Ga-PSMAPET/CT was performed: axial cuts of PET (**a**), fused PET/CT (**b**, **c**), and CT images (**d**) showing no evidence of radiotracer avid disease in the whole body notably in the prostate bed.

At least one malignant focus was found in 34/52 patients (65.4%) (Fig. 1), while 18 (34.6%) patients had a negative PSMA-PET/CT scan with no detectable disease (Fig. 2). We found a significant correlation between lesion identification and PSA mean at the time of imaging (p = 0.006). The detection rate was 2/4 (50%) for PSA < 0.2, 5/10 (50%) for PSA 0.2–0.49, 3/6 (50%) for PSA 0.5–0.99, 6/12 (50%) for PSA 1–1.99, 8/9 (88.9%) for PSA 2–3.99, and 10/11 (90.9%) for PSA 4–10. However, there was no statistically significant correlation between the imaging positivity and different PSA values at time of PSMA/PET (*p* = 0.09).

PSMA-PET/CT scans were positive in 32/45 patients (71.11%) whose PSAdt was ≤ 10 months versus 2/7 (28.6%) patients whose PSAdt was above 10 months.

PSA imaging results were significantly associated with PSAdt means (*p* = 0.006). However, it was not significantly associated with PSAdt when comparing ≤ 10 months to > 10 months (*p* = 0.08).

**Figure 3:**
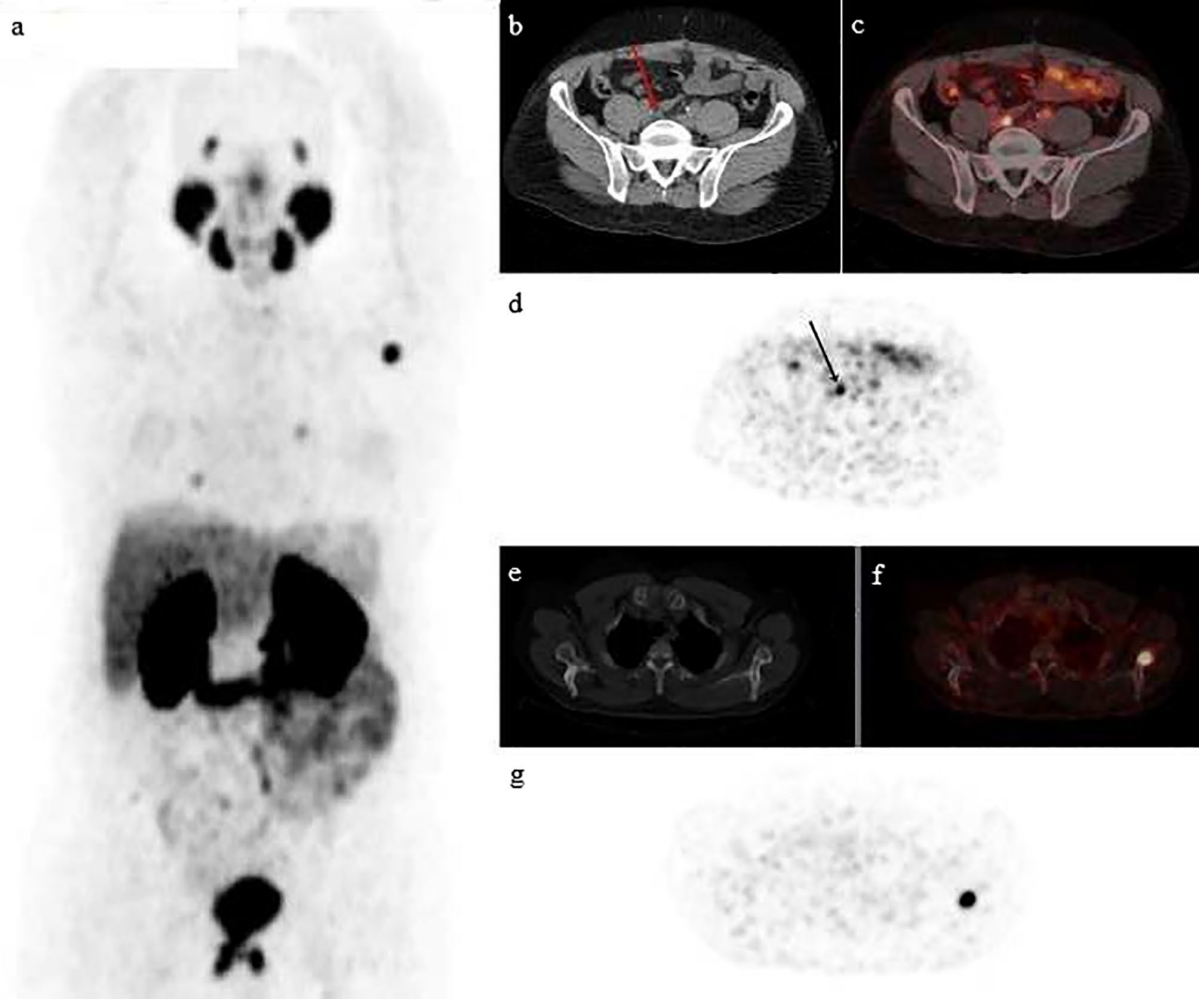
A 68-year-old patient status post radical prostatectomy. PSA level at time of acquisition: 9 ng/mL. Whole body 68 Ga-PSMAPET/CT was performed. Maximal intensity projection image (**a**) showing metastatic disease. Axial CT (**b**) showing disease in the right common iliac lymph node (red arrow). PET/CT (**c**) showing focal areas of radiotracer uptake in the apical region of the prostatic bed associated with pre-sacral radiotracer avid lymph node as well as multiple radiotracer avid bone lesions. PET image (**d**) shows focal uptake at the right common iliac node (black arrow). Axial thoracic CT (**e**) fused with PET (**f**) shows focal uptake in the left scapula. PET image (**g**) demonstrates this lesion.

Moreover, PSMA PET/CT was significantly associated with TNM stage (*p* = 0.03) (Fig. 3).

To note, GS was not reported in two out of 34 patients with positive PSMA PET/CT scans, as these patients performed their biopsies in other hospitals. Statistical analysis of 32 patients with positive imaging findings showed a significant correlation between PSMA PET/CT scan positivity and GS (*p* = 0.02). Furthermore, the detection rate was 1/3 (33.33%) in patients with GS 6, 17/29 (58.6%) in GS 7, 8/10 (80%) in GS 8 and 6/6 (100%) in GS 9. However, there was no statistically significant correlation between the positivity of the PSMA PET/CT scans and the different categories of GS (p = 0.09) (Table 3).

**Table 3:** Patients’ Gleason Score based on PSMA PET/CT results.

	PSMA-PET/CT negative	PSMA-PET/CT positive	*p*-value
	n = 18	n = 32	
Gleason	7 ± 0.5	7.6 ± 0.8	0.02
6	2 (12.5%)	1 (3.1%)	0.09
7	12 (75%)	17 (53.1%)	
8	2 (12.5%)	8 (25%)	
9	0 (0%)	6 (18.8%)	

In Table 4, multivariate logistic regression showed that GS was significantly associated with positive PSMA-PET/CT scans (OR: 5.94; 95% CI 1.25–28.38; *p* = 0.03).

**Table 4:** Association of clinical covariates with likelihood of detection by 68 Ga PSMA-PET/CT.

	Odds ratio	*P*-value	[95% Conf. Interval]	
Gleason score	5.94	0.03	1.25	28.38
PSA at PSMA-PET	2.54	0.02	1.15	5.63

Similarly, PSA values at the time of PSMA-PET/CT scans was also positively associated with imaging positivity (OR2.54; 95% CI 1.15–5.63; *p* = 0.02).

There was no significant association between PSMA PET/CT status and time to relapse (*p* = 0.21), ADT treatment (*p* = 0.08), or primary therapy (*p* = 0.46).

## Discussion

In our study, the positivity rate of PSMA PET/CT with at least one malignant focus was 65.4%, with similar rates ranging between 60–75% in other studies^22–25^. High PSA levels at the time of the PET/CT examinations were associated with higher PSMA PET/CT positivity, where a mean difference in PSA between positive and negative PSMA PET was found to be 4.5 ng/mL in one meta-analysis^26^. This is most likely due to the higher burden of disease with higher PSA levels^2^.

When it comes to positivity rates stratified by PSA levels, our study revealed equal or slightly higher overall PSMA PET/CT positivity per group when compared to the known literature: (PSA < 0.2: 50% vs 36.8%, PSA 0.2–0.49: 50% vs 43.3%, PSA 1–1.99: 50% vs 58.9%)^27^.

Features of aggressive or advanced disease were correlated with higher positivity rates, including shorter PSAdt (*p* = 0.006) and higher GS (*p* = 0.02). These findings were in concordance with the current literature, with PSAdt found to be an independent predictor of bone metastases and high GS associated with pelvic lymph node metastases^27,28^. This was predictable because shorter PSAdt and higher GS correlate with greater tumor extent and higher tumoral cells turnover, providing more sites for PSMA ligand binding.

The findings obtained from the logistic regression showed that the positivity of the PSMA-PET scans was associated with four main factors: PSA at the time of the PET/CT examination, PSAdt, GS, and TNM stage^29,30^. These findings were in concordance with the available literature on the value of PSMA PET/CT in evaluating patients with PCa with BCR^22,23,31–34^.

In our study, no significant association was detected between PSMA PET positivity and time to relapse. Previous literature has shown higher positivity of PSMA PET in shorter time to relapse when the time period was less than 29.5 months^35^. This may be explained by the relatively shorter follow up time in this prospective study (24 months), compared to the longer times in the abovementioned retrospective cohort^35^.

The use of ADT was not found to be associated with PSMA PET positivity, while some previous literature has shown a significant increase in the rates of positive PSMA PET in patients undergoing ADT^36^. In this study, 17/21 (80.9%) of patients who were on ADT had a positive PSMA PET/CT, while only 17/31 (54.8%) of patients who did not receive ADT had a positive PSMA PET/CT. No significance was detected (*p* = 0.08), which may be explained by our sample size.

This study showed no significant association between PSMA PET positivity and primary therapy in the setting of BCR (*p* = 0.46), such that 8/10 (80%) of patients who were on RT had a positive PSMA PET/CT, while only 26/42 (61.9%) of patients who underwent RP had a positive PSMA PET/CT. This is most likely due to the higher number of patients who received RP relative to RT in this cohort.

## Limitations

Three limitations were encountered in this study, the first being the use of histopathologic confirmation as a gold standard. This method of confirmation was not always feasible from an ethical and a practical point of view. Consequently, the constellation of known standard references were used, such as clinical and laboratory values, and drop in PSA levels after therapy. This is also the reason why the negative predictive value of ^68^GA PSMA PET/CT was not assessed.

The second major limitation encountered was the monocentric approach of the study, which is the cause of the relatively small sample size, bearing in mind that AUBMC is one of the biggest institutions and reference centers in the Middle East. The third limitation burdening this study is the inclusion of 4 patients with a PSA < 0.2 ng/mL. These patients were included due to the proximity of their PSA levels (0.18, 0.19 ng/mL) to the lower limit for inclusion, while also keeping note of the small sample size at our single center study. Nonetheless, this small study design was a minor step in confirming the capability of PSMA PET/CT in detection of local and metastatic recurrence in patients with BCR. Overall survival was not calculated due to the short follow up time (24 months) and the generally protracted course of disease in PCa, with all 52 patients surviving beyond the 24-month mark in this study. Larger multicenter studies with longer follow-up periods are needed for better investigation of subsequent changes in management.

## Conclusion

This single center prospective study on PSMA-PET/CT confirmed its ability and strength in detection of PCa recurrence in the setting of BCR. Our study with a limited number of patients showed promising results. The positivity of PSMA-PET/CT imaging was significantly associated with PSA at PET imaging time, PSAdt, GS, and TNM staging. PSMA- PET/CT is a highly promising modality in the work up of patients with PCa in the setting of BCR for earlier detection of disease recurrence.

## Data Availability

The datasets generated during and analysed during the current study are available from the corresponding author Dr. Mohamad Haidar on request.
